# Smartphone and web-based independent consultation and feedback for joint replacement surgeries: a randomized control trial protocol

**DOI:** 10.1186/s12911-021-01457-2

**Published:** 2021-03-05

**Authors:** Guixian Tong, Qingqing Geng, Tong Xu, Debin Wang, Tongzhu Liu

**Affiliations:** 1 School of Management, Hefei University of Technology, No.193 Tunxi Road, Hefei, People’s Republic of China; 2 The First Affiliated Hospital of USTC, Division of Life Sciences and Medicine, University of Science and Technology of China, No.17 Lujiang Road, Hefei, People’s Republic of China; 3 The First Affiliated Hospital of Anhui University of Traditional Chinese Medicine, Anhui University of Traditional Chinese Medicine, No.177 Meishan Road, Hefei, People’s Republic of China; 4 School of Data Science, University of Science and Technology of China, No. 443 Huangshan Road, Hefei, People’s Republic of China; 5 School of Health Service Management, Anhui Medical University, No.81 Meishan Road, Hefei, People’s Republic of China

**Keywords:** Medical materials, Clinical decisions, Consultation, Feedback, Joint replacement surgeries

## Abstract

**Background:**

Cost control and usage regulation of medical materials (MMs) are the practical issues that the government pays close attention to. Although it is well established that there is great potential to mobilize doctors and patients in participating MMs-related clinical decisions, few interventions adopt effective measures against specific behavioral deficiencies. This study aims at developing and validating an independent consultation and feedback system (ICFS) for optimizing clinical decisions on the use of MMs for inpatients needing joint replacement surgeries.

**Methods:**

Development of the research protocol is based on a problem or deficiency list derived on a trans-theoretical framework which incorporates including mainly soft systems-thinking, information asymmetry, crisis-coping, dual delegation and planned behavior. The intervention consists of two main components targeting at patients and doctors respectively. Each of the intervention ingredients is designed to tackle the doctor and patient-side problems with MMs using in joint replacement surgeries. The intervention arm receives 18 months' ICFS intervention program on the basis of the routine medical services; while the control arm, only the routine medical services. Implementation of the intervention is supported by an online platform established and maintained by the Quality Assurance Center for Medical Care in Anhui Province, a smartphone-based application program (APP) and a web-based clinical support system.

**Discussion:**

The implementation of this study is expected to significantly reduce the deficiencies and moral hazards in decision-making of MMs using through the output of economic, efficient, sustainable and easy-to-promote cooperative intervention programs, thus greatly reducing medical costs and standardizing medical behaviors.

***Trial registration number*:**

ISRCTN10152297.

## Background

With increasing demand for health services and rapid development of new medical technology worldwide, the dependence of clinical medical practice on medical materials (MMs) is growing [1]. High-value MMs account for more than half of total hospital MMs cost [2–4], extensive use of these MMs inevitably incurs great cost burden on hospitals and patients. More importantly, outside the scope of social medical insurance also means lack of third-party medical supervision [5–8]. In recent years, China government has enacted a series of policies focusing on the control of drug use and clinical diagnosis and treatment procedures, which make use of MMs an effective "alternative" for meeting certain policy requirements. For example, caped proportion of medication expense in overall cost is a widely adopted policy. Therefore, increased MMs use means decreased chance of bringing this policy [9, 10]. In order to address high MMs cost, China government has enacted a range measures such as "zero marks for medicines prescribed in hospitals", "centralizing procurement" and "minimum market relays". Some researchers have proposed "GPO (group purchasing organizations) procurement", "procurement alliance" and "zero inventory management". However, these measures have limited effect on curbing the rapid growth of MMs cost. These measures focus primarily on MMs supply with little attention being paid to decision-making process behind MMs use. Clinical decision-making happens in a complex and tricky context featuring medical uncertainty, information asymmetry and fragmented doctor–patient relationship and there are extensive variations and defects in doctors’ and patients’ response to these features [11–13]. Being the "dual delegate" for patients and the third-party payers, doctors are prone to various irrational conducts which are hardly detectable due to great information asymmetry, For example, doctors may order sophisticated examinations mainly for excluding some rare cases and thus reducing medical uncertainty and protecting themselves from credit loss or legal actions. Doctors may be selective in soliciting, recording and giving out information that leads to certain preferred clinical procedures [14–17]. By comparison, patients assume a much disadvantageous position in doctor–patient interactions. Patients are seldom allowed to convey detailed symptoms, history, fears and preferences in limited encounters with their doctors [18]. Patients lack medical knowledge for assessing the benefits and dis-benefits of different diagnosis and treatment procedures [19, 20]. In addition, patients often have various misperceptions of their doctors and vice versa [21, 22].

Contemporary interventions aimed at modifying clinical behavior focus primarily on education, information feedback, and reminders [23]. Many studies demonstrate that carefully designed continuing medical education (CME) courses can effectively improve doctors' use of diagnosis and treatment procedures [24–28]. With the rapid development of communication technology, the proportion of CME that uses the Internet (such as distance education, tele-consultation) is growing among young doctors. Interactive online technologies have proved to be able to change the way of medical education as well as clinical practice [29–32]. Although patients are disadvantageous as compared with doctors in terms of medical knowledge, they directly or indirectly affect clinical decisions in a variety of ways. Given that clinical decision-making is closely linked to patients’ health and expenses, there is great potential to mobilize patients in participating and optimizing clinical decisions [33]. Ringdal et al. reproted that promoting patient participation begins by understanding the patients’ unique preferences and needs for care, establishing a good relationship and paying attention to each patient’s ability to participate despite their illness [34]. D’Agostino and colleagues’ research results suggested that communication training is an effective approach to increase patients’ total level of active participation in healthcare interactions and that some communication behaviors may be more amenable to training (e.g., expressing concerns) [35].

## Study aims

This study aims primarily at developing and validating an independent consultation and feedback system (ICFS) for optimizing clinical decisions on use of MMs for inpatients needing joint replacement surgery. A secondary aim of this study is to conceive and refine a practical framework for identifying and tackling deficiencies in MMs-related decision-making on both doctor and patient side.

## Methods/design

### Study design and sampling

The study validates the effectiveness of the ICFS through a cluster randomized controlled trial (RCT) in Anhui, an inland province located in Eastern China with a population of 63.7 million and 1290 hospitals spread over 140,100 square kilometers. The RCT will strictly comply with the CONSORT guidelines.

A total of 30 hospitals will be recruited into the RCT. The sampling proceeds as the following: (1) list all the existing hospitals in Anhui Province that perform joint replacement surgery; (2) randomly select 30 non-adjacent hospitals (hospitals not in the same city) from the hospitals listed; (3) randomly allocate the selected 30 hospitals into "intervention group" and "control group"(in a 1:1 ratio using a computer-generated random number sequence by third-party statistician); (4) the departments of orthopedics in all hospitals of intervention and control groups are regarded as participating departments, and all doctors in these participating departments are regarded as participating doctors. The reason for selecting hospitals not located in the same city is to prevent cross-contamination between hospitals in intervention and control groups.

### Eligibility criteria of participants

The study recruits both orthopedist and their patients. All orthopedists from the selected hospitals are encouraged to participate in this study. The inclusion criteria of patients include: (1) inpatient patients awaiting hip or knee joint replacement surgery; (2) has the ability to communicate and make independent decisions; (3) not less than 18 years old.

### Sample size

The primary outcome variable was the cost of MMs, and the anticipated MMs cost reduction rate was 20% with an alpha level of 0.05 and 90% power. Considering the cluster sampling efficiency loss (about 30%), design effect (about 2.45) and attrition of participants (about 20%), we estimated a total sample size of 1100 patients, 550 in each group.

### Intervention

#### Guiding framework

**Fig. 1:**
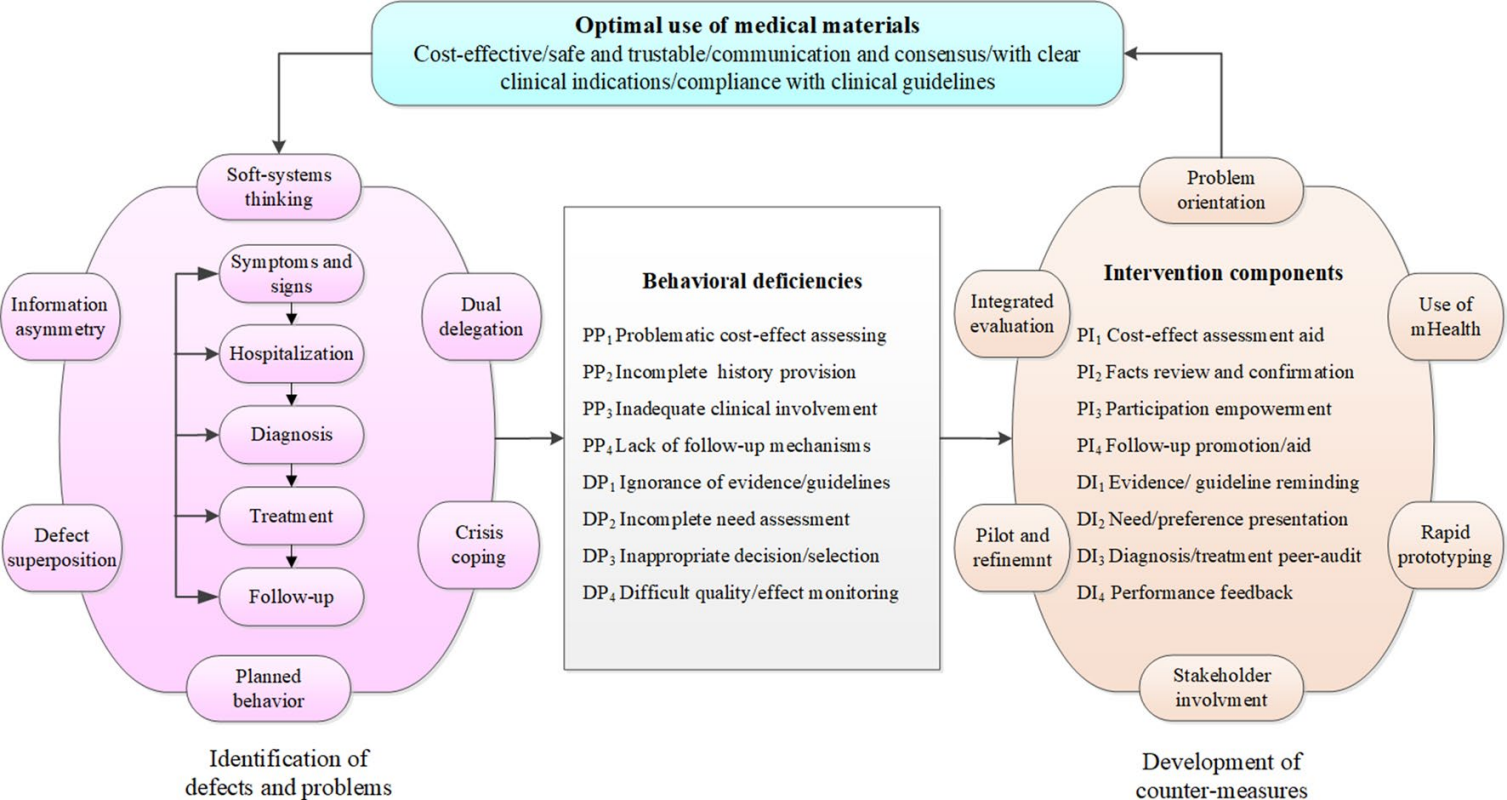
Framework of the study protocol. The research protocol framework includes two aspects including defects and problems identification (PP_1_–PP_4_ represent patient side problems, DP_1_–DP_4_ represent doctor side problems) and counter-measures development (PI_1_–PI_4_ represent patient intervention contents, DI_1_–DI_4_ represent doctor intervention contents)

Development of the research protocol is based on a problem or deficiency list derived on a trans-theoretical framework which incorporates including mainly soft systems-thinking, information asymmetry, crisis-coping, dual delegation and planned behavior (Fig. 1). Soft systems-thinking guides systematically unwinding the complex MMs decision system into relatively manageable critical processes starting from symptoms to admission, diagnosis, treatment and then follow up after hospitalization; while the other theories inform identification of potential problems for each of the processes derived above. For example, we performed process-by-process scrutiny of potential moral risks caused by information discrepancies between doctors, patients and third payers according to information asymmetry.

#### Problems list

Based on the abovementioned framework, we worked out a list of most important problems/deficiencies with existing MMs via qualitative interviews and consensus group activities involving relevant doctors, patients and third payer staff (Table 1). The list comprises problems/deficiencies from both doctor and patient sides. The doctor side problems (DPs) fall into four categories, i.e., problems regarding evidence and guidelines (DP_1_), demand and preference analysis (DP_2_), procedures and items selection (DP_3_) and quality and effect evaluation (DP_4_). Similarly, patient side problems (PPs) concern analysis and judgment (PP_1_), symptom and history providing (PP_2_), communication and decision-making participation (PP_3_) and follow-up management (PP_4_).

**Table 1:** Deficiencies with existing medical materials use

Code and title	Short description of deficiencies
DP_1_: Ignorance of evidence/guidelines	Doctors do not understand the latest research evidence and regulations regarding medical implants using (DP1a)
	Doctors do not have enough time in studying the latest professional development and progress owing to the heavy clinical works (DP1b)
	Doctor face difficulties to identify and abstract the latest useful evidence from numerous and rapidly growing literatures (DP1c)
	Doctors lack adequate foreign language proficiency contrasted by that the majority of medical research are published in English (DP1d)
DP_2_: Incomplete need/preference assessment	Doctors tend to conduct systematic and sophisticated auxiliary examinations for collecting evidence to protect doctors/hospitals from low probability "clinical accidents", rather than to improve the accuracy of diagnosis and treatment (DP2a)
	Doctors are keen at looking for physical determinants of diseases but pay less attention to psychological factors, family economic status and cost-effectiveness (DP2b)
	Doctors lack the ability of systematic analysis and multi-objective collaboration for patients’ needs and preferences (DP2c)
DP_3_: Inappropriate decision/selection	Doctors tend to order clinical diagnosis and treatment procedures (especially the MMs using) without clear indications (DP3a)
	Doctors often order a same and complete set of laboratory and imaging examinations to all their patients rather than selecting specific items according to the needs of specific patients (DP3b)
	Doctors tend to overuse specific MMs to cater to perceived demands of patients or sales agents (DP3c)
	Doctors tend to prescribe more or less certain MMs for demonstrating application of new technologies or to compliance to policy/management system requirements (DP3d)
	Doctors are often biased in selecting and recording patient symptoms and medical history, so as to justify application of specific diagnostic and therapeutic pathway and procedures (DP3e)
DP_4_: Difficult quality/effect monitoring	Lack of effective follow-up mechanism (DP4a)
	Lack of operable follow-up indicators and procedures (DP4b)
	Lack of adequate follow-up resources and technologies (DP4c)
PP_1_: Problematic cost-effect assessing	Patients can hardly obtain complete, objective and understandable information regarding treatment procedures and MMs use (PP1a)
	Patients do not have enough knowledge of relevant physiological/pathological mechanisms of disease development and thus lack the ability of independently comparing alternative procedures and making optimal decisions (PP1b)
	Patients and relatives have many irrational thinking patterns, such as "do everything one for health at any cost" (PP1c)
	Patients are often given misleading information about prognosis and diagnosis and treatment alternatives due to various reasons (PP1d)
PP_2_: Incomplete history provision	Patients often fail to fully recognize the importance of telling their doctors accurate history and symptoms (PP2a)
	Patients tend to conceal certain symptoms/history because of the presence of specific relatives or friends (PP2b)
	Patients may intentionally over-report/exaggerate some symptoms/history in order to obtain specific priority (such as earlier surgery) or compensation (e.g., medical insurance compensation) (PP2c)
	Patients may give biased report about their conditions to meet perceived expectations of doctors, relatives and friends (PP2d)
	Patients often lack the opportunity to check, revise and supplement symptoms and medical history collected in medical records (PP2e)
PP_3_: Inadequate clinical involvement	Patients have various suspicions and misunderstandings towards doctors and hospitals due to inaccurate self-media reports about negative cases (PP3a)
	Patients tend to "say good words in front of doctors " due to the influence of traditional Chinese culture (PP3b)
	Patients often avoid raising objections to doctors’ recommendations for diagnosis and treatment in order for fearing of upsetting doctors (PP3c)
PP_4_: Lack of follow-up mechanisms	Patients often do not keep in contact with the responsible doctors after their hospitalization (PP4a)
	Patients often have insufficient understanding of family rehabilitation and self-management after hospitalization (PP4b)
	Patients often seek for health services from different hospitals rather than a same hospital, which decreased the consistency/continuity of diagnosis, treatment and evaluation (PP4c)

#### Intervention ingredients

The intervention consists of two main components targeting at patients and doctors respectively. For patients, the intervention promotes option assessment (PI_1_), facts review and confirmation (PI_2_), self-expression (PI_3_) and follow-up management (PI_4_). For doctors, the intervention facilitates evidence promotion (DI_1_), demand feedback (DI_2_), procedure audit (DI_3_) and performance comparison (DI_4_). Each of these intervention ingredients is designed to tackle the doctor and patient-side problems with existing MMs as derived above. Taking the example of option assessment (PI_1_), it is designed to empower patients and/or their relatives to assess the benefits, dis-benefits and preferences over alternatives of MMs use. This is reached by providing: (a) all treatment options (especially MMs options) and explaining the benefits, dis-benefits and application indicators of each options; (b) tailored questions and answers regarding their current diseases and treatment and MMs use options; (c) structured "cost-utility" evaluation scale to help patients systematically evaluate the effects of different joint replacement and MMs use options on their physiological, psychological and social functions; and (d) concise and easily understandable "cost-utility" evaluation summaries of optimum matching scheme suitable for their own and the corresponding reasons. The target problems and main contents and procedures of each of the intervention ingredients are summarized in Table 2.

**Table 2:** Summary intervention ingredients

Code and title	Short description of interventions
PI_1_: Cost–benefit evaluation aid	
Objectives	Help patients make a relatively systematic evaluation on the alternative diagnosis and treatment procedures including MMs use
Interventions	Display, to patient, diagnosis/treatment alternatives (especially options of MMs use) and corresponding advantages, disadvantages and indications
	Provide problem-solving cases so as to eliminate the problems and doubts that patients may have regarding their current conditions and MMs use
	Provide structured "cost-utility" rating scale to help patients systematically evaluate different joint replacement procedures/MMs on their physiological, psychological and social functions
	Provide easy-to-understand "cost-utility" evaluation summaries to help comparison and selection of different treatment/MMs options
PI_2_: Facts review and confirmation	
Objectives	Enable patients to review, confirm and supplement disease symptoms and history recorded by the doctor so as to correct and prevent selection biases and mistakes
Interventions	Remind patient of the importance of reporting and recording accurate symptoms and medical history
	Provide relatively "private", "independent" and convenient environment for patients to check and supplement information of symptoms and medical history without being affected by relatives, friends, doctors, etc.
	Ask patients to perform at least two times of "facts review and confirmation", one before treatment and MMs use and the other 1 week after discharge from hospital
PI_3_: Participation empowerment	
Objectives	Provide patients (via mobile app/personalized web page) with precautions, tips and skills in communicating with doctors
Interventions	Tell patients common misunderstandings and misperceptions about clinical practices and doctors/hospitals
	Tell patients the importance of fully and accurately reporting to doctors their conditions, especially the experience that doctors can not feel
	Tell patients that they can ask doctors, if they want, to discussion their health conditions and treatment in a private space free from potential disturbances
	Tell patients that it is their own interest to correct mistakes and fill gaps in the symptoms and medical history as recorded in their clinical records; Tell patients that they need to straightforwardly speak out their preferences, expectations and difficulties in selecting diagnosis and treatment options
PI_4_: Follow-up promotion/aid	
Objectives	Encourage and help patients practice self-care management after discharge and cooperate in follow-up visits and evaluation feedbacks
Interventions	Tell and encourage patients, via a daily text message for 1 month after discharge, how to practice post operation rehabilitation and other self-care activities
	Ask patients questions about their satisfaction and effectiveness of the previous inpatient care and problems encountered after the discharge
	Give feedback, via the daily text message again, to patients on the follow-up query and in particular, on helping them solve problems encountered
DI_1_: Evidence/guideline reminding	
Objectives	Improve practice quality and compliance with relevant policies, professional guidelines and use of research evidences
Interventions	Post bulleted key requirements of relevant policy/guideline together with hyperlinks to detailed files of these policy/guidelines on the homepage of the clinical support system to be described later
	Display a pop-up window containing indications or view points for doctors to check and confirm their decisions on material use
	Maintain a bimonthly briefing of newly published evidences in relevant fields on the aforementioned homepage
DI_2_: Need/preference presentation	
Objectives	Help doctors better understand their patient’s needs, expectations and preferences
Interventions	Present doctors with a brief report summarizing the main points of medical record that have been confirmed, amended and complemented by the patients
	Present doctors with a list of the preference ratings of potential treatment options (including options of MMs use) given by a specific patient under concern
DI_3_: Diagnosis/treatment peer-audit	
Objectives	Introduce a pragmatic mechanism of learning from real cases and technical assistance between peer doctors
Interventions	Form a peer-review group consisting of mainly the participating doctors themselves and a few well-known local experts from outside the study participants
	Perform a monthly “cross-review”, in which two patients were randomly selected as the review cases for each participating doctor from all the patients he/she has served in the past month and then reviewed anonymously by one of the other peer group members
	Email the results of the above peer review (comments and suggestions regarding the two selected cases) to the corresponding doctor
DI_4_: Performance feedback	
Objectives	Give doctors a bimonthly feedback and encourage good performance and inform identification and overcome of potential problems/shortcomings
Interventions	Form a set of agreed performance indicators by involving all the participating doctors in the intervention group
	Post top 10 performance stars on the homepage of APP for patients and on the homepage of the support system for doctors, and update it every 2 months
	Send a doctor-specific feedback email, again bimonthly, to each participating doctors in the intervention group

#### Electronic support

Implementation of the intervention is supported by an online platform established and maintained by the Quality Assurance Center for Medical Care in Anhui Province, a smartphone-based application program (APP) and a web-based clinical support system (CSS). The CSS is designed for use by doctors; while the APP, for patients.

### Control

The participants in the control group receive the existing medical services without any additional consultation and feedback services adopted in the intervention group.

### Study and data integrity

For the integrity of study and data, the study design follows the CONSORT (Consolidated Standards of Reporting Trials) statement.

### Measures

As shown in Table 3, the primary outcome measures for evaluating the intervention include: (1) Cost of medical materials assessed using questionnaire custom-designed for this study at 6 months and 12 months after discharge, these data will be used to calculated cost-effectiveness; (2) Quality-of-life assessed using the EQ-5D-5L scale at discharge, and 6 months, 12 months and 18 months after discharge. The secondary outcome measures include: (1) Peer expert audit score assessed using a custom-designed questionnaire at 6 months after discharge; (2) Quality control inspection score assessed using a custom-designed questionnaire at 6 months after discharge; (3) Patient awareness rate (treatment schedule, surgical risk, material selection, etc.) assessed using a custom-designed questionnaire at discharge, and 6 months, 12 months and 18 months after discharge; (4) Patient satisfaction assessed using a custom-designed questionnaire at discharge, and 6 months, 18 months after discharge; (5) Total medical expense assessed using a custom-designed questionnaire at 6 months and 18 months after discharge.

**Table 3:** Variables and time points for outcome measures

Measure	Time point (month after discharge)			
	Discharge day	6 months	12 months	18 months
Cost of medical materials		√		√
Quality-of-life	√	√	√	√
Peer expert audit score		√		
Quality control inspection score		√		
Patient awareness rate	√	√	√	√
Patient satisfaction	√	√		√
Total medical expense		√		√

### Data collection and analysis

The data for intervention evaluation will be collected from two sources: patients' medical records retained by the participating hospitals (e.g., electronic medical records, paper treatment records and MMs charge records) and patients' baseline and follow-up health related information via multi-stage surveys. For medical records, we first periodically centralized copy electronic documents and borrow paper documents from hospital, and then we arrange two uniformly trained graduate students (each hospital) independently extract data according to the pre-designed structured "data table". When inconsistencies arise, they negotiate to reach a consensus. The baseline and follow-up surveys will be conducted by uniformly trained graduate students in the form of telephone interviews with structured questionnaires. The follow-up strives to reach less than 20% attrition of participants by means of at least 3 times of reminding messages and 5 times of telephone calls at different time and days. The researchers are trained to record reasons of attrition for each of the patients they have lost. Characteristics of patients lost to follow-up will be analyzed and compared to those who finish the interventions to assess potential attrition biases. Data analysis will focus mainly on comparing the differences in overall and specific indicators of appropriateness of joint replacement surgery and MMs used between the intervention and control arms.

## Discussion

The “independent consultation-feedback mechanism” being tested has several advantages compared with traditional clinical behavioral interventions. Firstly, this intervention considers the impact of doctors, patients and doctor–patient communications, rather than single doctor factor, on the treatment scheme selection and MMs use. In clinical activities, doctors are in the dominant position of information because of their knowledge of medicine and usually make decisions as agent instead of patients. Although patients are in the disadvantaged position of information due to lack of medical knowledge and medical equipment, they have their own information advantages regarding symptoms, medical history and treatment preference, etc., which are essential components in designing clinical treatment schemes. The asymmetrical information between doctors and patients initiates the moral risk easily, which gives birth to high medical costs and non-optimal therapeutic effects. Past years witnessed several researches investigating the influence of doctor interventions (e.g., education, information feedback and reminders) on the clinical decisions and behaviors, yet studies explored the cooperative role of doctor–patient interaction are generally lacking.

Secondly, the scheme adopts individualized intervention measures against specific behavioral defects. Clinical decisions of joint replacement surgeries are influenced by various factors in terms of communication strategy using, professional knowledge lacking, therapeutic items selection and medical plan preference, etc., which make it hard for traditional single-factor interventions and comprehensive interventions to identify key behavioral defects yield significant effects. In order to properly tackle this challenge, we adopt soft systems thinking which originates from software development and views all elements in an interactive and holistic way. We analyze the basic links of the cost of joint replacement surgeries, the main problems that may occur in each link and the appropriate interventions for each problem. At first, based on literature review, our previous researches, relevant clinical guidelines and follow-up observations, we systematically decomposed the factors affecting the consumption of MMs in complex joint replacement surgeries into several relatively simple basic links. Then we explore and list the possible problems and shortcomings in each link through literature review and insider interviews, depends on which we develop as many interventions as possible to overcome and eliminate the ultimate identified "problems and shortcomings". At last, according to the overall objective of consumption optimization, we refined limited number of relatively effective interventions ("short list") from the raw "long list" of interventions produced by the above-mentioned process. The "short list" should avoid possible "equivalence" as far as possible when fully pursuing the role of "synergy" and "emergence" among the measures.

Thirdly, all interventions will be performed via a unified third party consultation and feedback platform, that is to say, a single independent platform be used to meet the service needs of all included patients and doctors. The so-called "independent" consultation feedback mechanism refers to the consultation feedback service provided by a third party (rather than the hospital in which the patient is hospitalized). The reason for constructing this platform including: (1) information exchange between doctors and patients involves many aspects including data collection, matching and pushing, etc., the workload is too huge and difficult to achieve if these information are processed manually; (2) the platform can realize the evaluation and feedback of patients, doctors and third-party evaluation agencies by effectively analyzing the interactive records and treatment behaviors, so as to assist decision-making of all parties, improve the efficiency of diagnosis and treatment, as well as to reduce the subjectivity of evaluations, etc.; and (3) third-party platform can effectively reduce the subjective evaluation results with individual tendency caused by interest factors.

## Limitations

Our research also has limitations. Although our intervention is designed in accordance to doctor and patient side deficiencies with existing MMs use, considering the short hospitalization time of orthopedic patients and the complexity of personalized scales of cognitive-affection personality system, we did not design and implement personalized interventions for patients with different personality characteristics, which may to some extent affected the effectiveness of interventions. In addition, in our study, we considered only the influence of doctor, patient and doctor–patient relationship on clinical decision-making, health policy and hospital management system may also play important role in the selection of treatment plans and MMs in clinical activities and these factors are closely related to patient and doctor's behaviors. Future researches should incorporate these factors into consideration and construct more comprehensive multi-variable intervention system.

## Data Availability

The data used and/or analyzed during the current study available from the corresponding author on reasonable request.

## Abbreviations

MMs: Medical materials
APP: Application program
CSS: Clinical support system
ICFS: Independent consultation and feedback system
GPO: Group purchasing organizations
CME: Continuing medical education
RCT: Randomized controlled trial
DPs: Doctor side problems
PPs: Patient side problems
CONSORT: Consolidated Standards of Reporting Trials
